# Triboelectric Nanogenerator-Embedded Intelligent Self-Aligning Roller Bearing with the Capability of Self-Sensing, Monitoring, and Fault Diagnosis

**DOI:** 10.3390/s24237618

**Published:** 2024-11-28

**Authors:** Hao Shen, Yufan Lv, Yun Kong, Qinkai Han, Ke Chen, Zhibo Geng, Mingming Dong, Fulei Chu

**Affiliations:** 1 School of Mechanical Engineering, Beijing Institute of Technology, Beijing 100081, China; s2148509@ed.ac.uk (H.S.); 3120240319@bit.edu.cn (Y.L.); cukie001@163.com (K.C.); vdmm@bit.edu.cn (M.D.); 2 School of Engineering, University of Edinburgh, Edinburgh EH9 3FB, UK; 3 Tangshan Research Institute, Beijing Institute of Technology, Tangshan 063015, China; 4 State Key Laboratory of Mechanical Transmission for Advanced Equipment, Chongqing University, Chongqing 400044, China; 5 Department of Mechanical Engineering, Tsinghua University, Beijing 100084, China; hanqinkai@tsinghua.edu.cn (Q.H.); chufl@mail.tsinghua.edu.cn (F.C.); 6 Inner Mongolia First Machinery Group Co., Ltd., Baotou 014032, China; 7 School of Aeronautics and Astronautics, University of Electronic Science and technology of China, Chengdu 611731, China; gzbgeng@uestc.edu.cn

**Keywords:** triboelectric nanogenerator, intelligent bearing, speed sensing, bias angle monitoring, fault diagnosis, deep learning

## Abstract

Monitoring the dynamic behaviors of self-aligning roller bearings (SABs) is vital to guarantee the stability of various mechanical systems. This study presents a novel self-powered, intelligent, and self-aligning roller bearing (I-SAB) with which to monitor rotational speeds and bias angles; it also has an application in fault diagnosis. The designed I-SAB is compactly embedded with a novel sweep-type triboelectric nanogenerator (TENG). The TENG is realized within the proposed I-SAB using a comb–finger electrode pair and a flannelette triboelectric layer. A floating, sweeping, and freestanding mode is utilized, which can prevent collisions and considerably enhance the operational life of the embedded TENG. Experiments are subsequently conducted to optimize the output performance and sensing sensitivity of the proposed I-SAB. The results of a speed-sensing experiment show that the characteristic frequencies of triboelectric current and voltage signals are both perfectly proportional to the rotational speed, indicating that the designed I-SAB has the self-sensing capability for rotational speed. Additionally, as both the bias angle and rotational speed of the SAB increase, the envelope amplitudes of the triboelectric voltage signals generated by the I-SAB rise at a rate of 0.0057 V·deg^−1^·rpm^−1^. To further demonstrate the effectiveness of the triboelectric signals emitted from the designed I-SAB in terms of self-powered fault diagnosis, a Multi-Scale Discrimination Network (MSDN), based on the ResNet18 architecture, is proposed in order to classify the various fault conditions of the SAB. Using the triboelectric voltage and current signals emitted from the designed I-SAB as inputs, the proposed MSDN model yields excellent average diagnosis accuracies of 99.8% and 99.1%, respectively, indicating its potential for self-powered fault diagnosis.

## 1. Introduction

Self-aligning roller bearings (SABs) are designed to withstand significant radial loads and relatively low axial loads, making them ideal for various applications, including in wind turbines, construction machinery, and power transmission systems. SABs can withstand axial loads, even when the shaft and the bearing housing are misaligned, enabling them to operate efficiently when there is a bias angle between their inner and outer rings. The bias angle results in a change in the contact angle, and the value of the change is called the self-aligning contact (SAC) angle [1]. An overweight SAC angle leads to weakened stiffness and intensified friction and internal clearance, thus shortening the operational life of the SAB [2,3,4]. Furthermore, SABs are prone to various faults due to the harsh working environment [5]. To avoid industrial disasters caused by mechanical faults, it is necessary to develop an efficient way of monitoring the health condition of SABs. Traditionally, the bias angle of SABs is measured with a coordinate measuring machine, which is expensive and cannot be used during real-time operations. Moreover, vibration-based fault diagnosis techniques usually require extra radial space for the installation of acceleration sensors. Therefore, there is a significant need to develop a self-powered and self-sensing method to monitor the dynamic behaviors and health conditions of SABs.

Triboelectric nanogenerators (TENGs) have recently provided an elegant and promising solution to the self-sensing monitoring tasks of mechanical systems. Based on the periodic transfer of triboelectric charges during mechanical motion, TENGs can convert mechanical energy into electrical energy at a certain power density [6]. TENGs have attracted considerable attention in the field of energy harvesting and self-powered sensing owing to the advantages of high structural integration, small volume, and low cost [7,8,9,10,11,12]. Self-powered sensors based on TENGs can be manufactured with a small size [13] and are capable of performing self-sensing tasks within a limited space. Meng et al. developed a TENG-based self-powered sensor that is integrated into glasses and the brake and accelerator pedals of a car, aiming to monitor driver behaviors [14]. Jin et al. implemented a wireless sensor based on a TENG and embedded it into a train chassis to monitor vibrations during the running process [15]. Xie et al. designed a novel TENG by pasting electrode and triboelectric layers onto gear teeth and carried out online fault monitoring using TENG triboelectric signals and a deep learning model [16]. Being sensitive to many physical quantities, TENGs are widely applied in mechanical systems for acceleration, distance, and speed sensing. Liu et al. observed that the output amplitude of a contact–separation TENG is proportional to the triboelectric–electrode displacement, which led to the design of a self-powered acceleration sensor for vibration monitoring [17]. Similarly, Pang et al. combined three TENG sensors for 3D acceleration monitoring [18]. Owing to their high integration degree, flexibility, and great sensitivity, TENG sensors have also been widely applied in self-powered intelligent bearings for the surveillance of rotational speed, slip rate, and health conditions. Gao et al. used the spherical ceramic rollers of bearings as a triboelectric material and developed an embedded TENG inside the bearing without causing damage, achieving real-time behavior monitoring [19]. Moreover, the authors proposed a compact intelligent bearing for skidding rate monitoring using an embedded freestanding-mode TENG [20]. Xie et al. studied the influence of triboelectric–electrode separation on the TENG output performance and proposed a long-life intelligent bearing with non-contact triboelectric layers, which was applied to monitor the speed and skidding rate of bearings [21]. Jiang et al. proposed a compact-mode self-powered smart bearing [22] and a smart bearing embedded with a membrane-based disk-type TENG [23], both of which were verified as being capable of fault diagnosis. The experiments showed that diagnostic accuracy could reach 90%, demonstrating that TENG-based smart bearings are promising for self-powered and self-sensing condition monitoring.

Though TENG-based smart bearings have shown considerable potential for self-powered and self-sensing condition-monitoring applications, there remains a research gap between intelligent bearings embedded with TENGs and the capability of simultaneously monitoring the dynamic behaviors and health conditions of SABs. To this end, we propose a novel-type self-powered intelligent SAB embedded with a TENG (I-SAB) in this study and verify its applications in self-powered rotational speed sensing, bias angle monitoring, and fault diagnosis. Specifically, a compact prototype of the I-SAB based on freestanding-mode triboelectric layers and a comb–electrode pair is fabricated. Three triboelectric layers are attached to an acrylic disk, which is fastened to the inner ring of the bearing using a nut. The disk, coated with triboelectric layers, serves as the rotor part of the TENG since it rotates simultaneously with the inner ring. Steel cushions are placed between the inner ring and acrylic disk in order to control the dielectric–electrode separation. Pairs tin electrodes with comb shapes are printed on a ring-shaped PCB via the hot-air solder leveling (HASL) process. The PCB is inlaid in acrylic housing that is adhered to the outer ring using neodymium magnets. Based on the designed I-SAB prototype, detailed design parameters, including the triboelectric layer material, comb–finger pair number, the distance between the electrode and triboelectric layer surface, and the triboelectric layer number, are discussed. Moreover, the variations in the triboelectric voltage and current with load resistance are measured to evaluate the output power of the I-SAB. The characteristic frequencies of the triboelectric current and voltage acquired at different speeds are validated as accurate indicators of rotational speeds. A test bench is constructed based on optical systems to conduct experiments on the I-SAB from various bias angles. Bias angle monitoring is performed by analyzing the envelope amplitude of the triboelectric voltage signals emitted from the I-SAB. A strong linear relationship between the triboelectric voltage envelope amplitude and bias angle is discovered; thus, the newly designed I-SAB is found to be reliable in bias angle monitoring. The triboelectric voltage and current signals emitted from I-SABs under different health conditions are also assessed and analyzed. We propose a novel Multi-Scale Discrimination Network (MSDN), based on the ResNet18 architecture, to extract and classify the weak fault features hidden in the signals emitted from the designed I-SAB with various faults. Both the triboelectric current and voltage signals emitted from the I-SABs are processed and used to train and evaluate the MSDN. The evaluation results of fault diagnosis show that the MSDN model trained using the triboelectric voltage and current signals emitted from the I-SAB achieves average diagnostic accuracies of 99.8% and 99.1%, respectively, outperforming the classical convolutional neural network (CNN) and ResNet18.

The main contributions of this study can be summarized as follows: firstly, we designed the first compact TENG that can be embedded to create an intelligent SAB with excellent self-powered rotational speed sensing and bias angle monitoring; secondly, with the aid of deep learning, we developed a novel MSDN model and achieved self-sensing fault diagnosis with superior diagnostic accuracy based on weak fault features.

## 2. Design and Implementation of I-SAB

### 2.1. Structure and Principle of I-SAB

**Figure 1:**
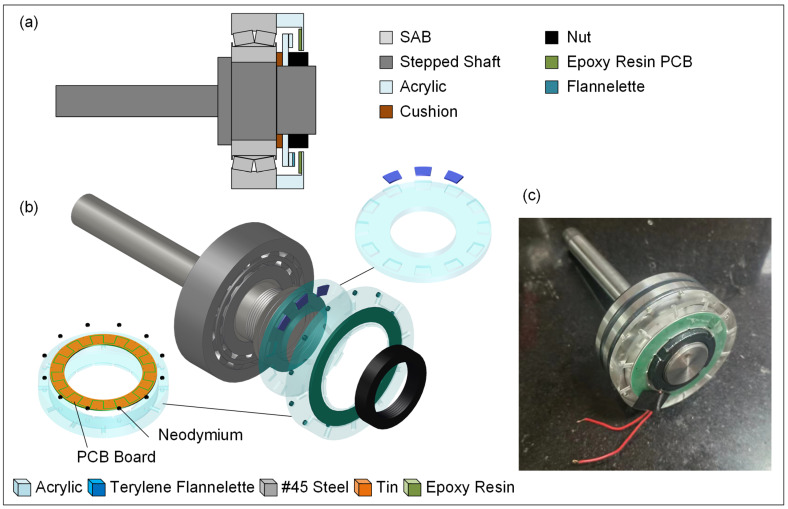
(**a**) Sectional view of I-SAB; (**b**) explosion diagram of I-SAB; (**c**) I-SAB prototype.

The sectional view of the designed I-SAB, which comprises an SAB (B21308CA/W33 type), an acrylic disk, acrylic housing, an epoxy resin PCB board, and cushions, is illustrated in Figure 1a. The designed I-SAB is embedded with a compact TENG, which is configured with an acrylic disk as the rotor and an epoxy resin PCB as the stator. Three flannelette layers, pasted on three adjacent bosses present on the disk, serve as the triboelectric layers of the TENG; meanwhile, a 12-tooth tin comb–electrode pair sprayed on the epoxy resin PCB forms the TENG electrode. The acrylic disk, acrylic housing, and 0.5 mm thick #45 steel cushions are processed by CNC machining. A pair of tin electrodes, approximately 2 μm thick, are created on a 1.5 mm thick PCB board using the hot-air leveling process. The PCB is fixed to the inner surface of the housing. The I-SAB is fixed onto the #45 steel stepped shaft and fastened with a standard M35 nut. The assembly process is illustrated in the explosion diagram in Figure 1b. Firstly, we inlaid the PCB and neodymium magnets into the acrylic housing; secondly, we threaded the bearing, the cushions, and the acrylic disk onto the stepped shaft; and thirdly, we screwed the tightening nut to fix the disk onto the inner ring of the SAB. Finally, we magnetically adhered the acrylic housing to the outer ring of the SAB. The I-SAB prototype is illustrated in Figure 1c.

**Figure 2:**
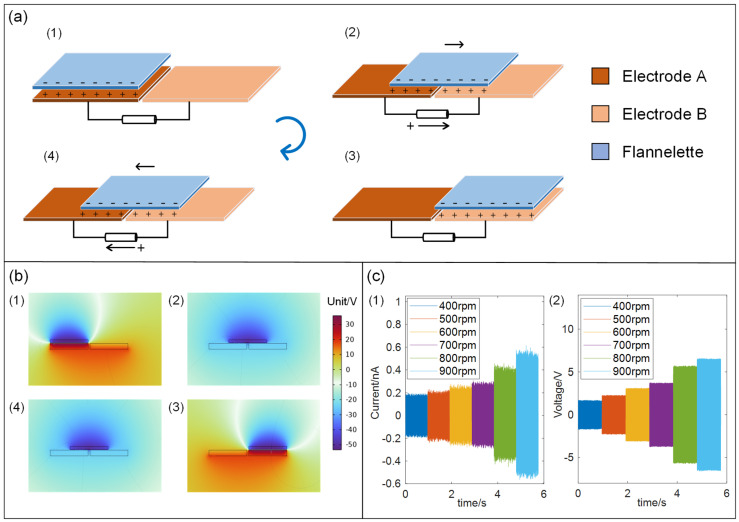
(**a**) Power generation mechanism of I-SAB; (**b**) simulation results of electrostatic field using COMSOL; (**c**) (1) short-circuit current and (2) open-circuit voltage from I-SAB under different rotational speeds.

The full working cycle for the TENG embedded into the I-SAB is illustrated in Figure 2a. As the shaft rotates, the flannelette triboelectric layers on the acrylic disk sweep along the circumferential direction of the comb fingers on the electrodes. A constant negative charge accumulates on the surface of the triboelectric layer. As the triboelectric layer moves between the combs of the different electrodes, electrons are periodically driven between the electrodes by the electrostatic forces. Initially, the triboelectric layer faces electrode A, whose negative charges cause an equal number of positive charges to accumulate on the electrode. In this state, as shown in Figure 2(a1), electrostatic equilibrium is established, and no charge transfer occurs between the two electrodes; thus, no current is observed. As the acrylic disk rotates along with the inner ring of the SAB, the triboelectric layer moves toward electrode B. Simultaneously, some positive charges transfer from electrode A to electrode B through the load circuit between the two electrodes. The transfer of charge between the electrodes results in there being a detectable transient current in the same direction, as shown in Figure 2(a2). When the triboelectric layer aligns with electrode B, a new electrostatic equilibrium is established, with positive charges accumulating on electrode B, as shown in Figure 2(a3). Then, the triboelectric layer sweeps back toward electrode A, causing the positive charges to transfer back to electrode A, as shown in Figure 2(a4). After the flannelette triboelectric layer moves back to the initial state, one complete cycle of power generation finishes. The continuous movements of the triboelectric layer repeat these stages (a1–a4), producing a continuous alternating triboelectric current (AC) signal between the two electrodes in the TENG. The open-circuit potential distribution corresponding to the four stages of one power generation cycle is shown in the image in Figure 2b, which was obtained using the finite element method (FEM) with COMSOL. The simulation results confirmed the power generation mechanism of the TENG inside the I-SAB, thereby demonstrating that an AC signal can be generated between the two electrodes of the TENG within the I-SAB when the inner ring of the SAB rotates. The short-circuit current and open-circuit voltage of the I-SAB under different speeds are acquired and are displayed in Figure 2(c1) and Figure 2(c2), respectively. It can be observed that both the peak current and the peak voltage increase from 0.2 nA and ±1.7 V to ±0.55 nA and ±6.2 V, respectively, with the rotational speed increasing from 400 rpm to 900 rpm.

### 2.2. Output Characteristics and Parameter Optimization

**Figure 3:**
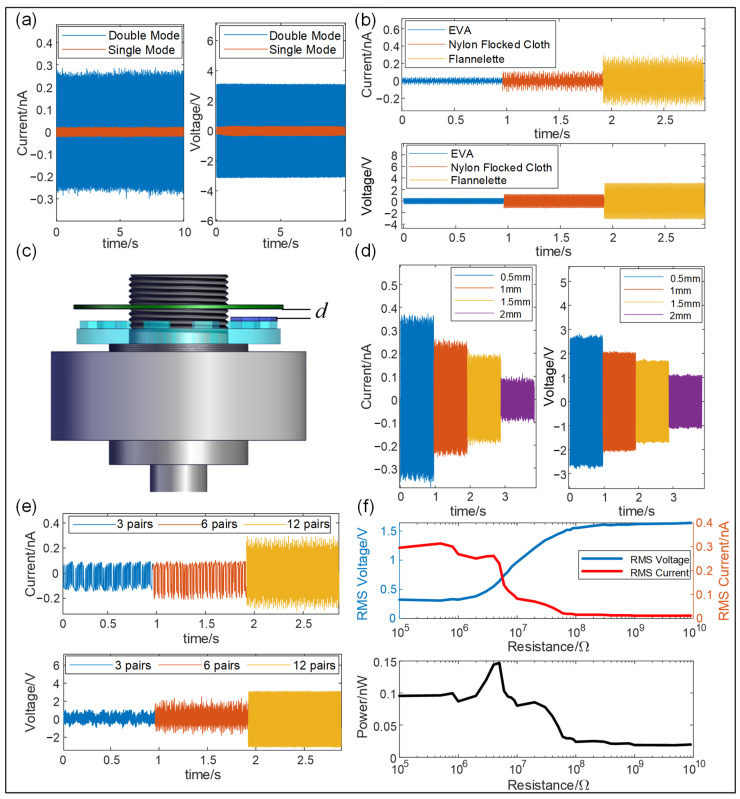
The output performance of the I-SAB with different design parameters. (**a**) The short-circuit current and open-circuit voltage in two working modes; (**b**) the short-circuit current and open-circuit voltage for different dielectric materials; (**c**) an illustration of the separation distance in the I-SAB; (**d**) a short-circuit current and open-circuit voltage with different separation distances; (**e**) a short-circuit current and open-circuit voltage with different comb–finger numbers; (**f**) variation in output power as load resistance changes.

To optimize the structural design of the I-SAB, we conducted the following experiments to examine the impact of structural parameters on the output characteristics of the TENG. Firstly, we sampled the output performance of TENGs in two different working modes, namely, a single-electrode mode and double-electrode mode. Figure 3a shows a comparison of the short-circuit current and open-circuit voltage of the I-SAB embedded with TENGs in two working modes. The results indicated that the short-circuit current and open-circuit voltage of TENG in the double-electrode mode reached peak-to-peak values of approximately 2.7 nA and 3 V, respectively, which are nearly 20 times and 10 times greater than those obtained in the single-electrode mode. For the single-electrode mode, the charge only accumulated at one electrode; thus, no additional charge gathered when the triboelectric layer moved away from the electrode comb. As a result, the electrode was only charged during half of the cycle, limiting the charge density and, consequently, reducing the output. This finding supports the selection of a double-electrode mode for our I-SAB design. Secondly, to identify the most suitable material for the triboelectric layer of the TENG in our I-SAB, we selected three different materials—flannelette, nylon-flocked cloth, and EPE—and acquired the triboelectric current and voltage at a rotational speed of 600 rpm. As shown in Figure 3b, the flannelette produced the highest short-circuit current and open-circuit voltage, with peak-to-peak values of 0.3 nA and 3.1 V, respectively. Based on its superior output performance, flannelette was selected as the triboelectric layer material in this study. Thirdly, another crucial design parameter, namely, the separation distance (as illustrated in Figure 3c) between the triboelectric layer and electrode, was optimized. The short-circuit current and open-circuit voltage from the I-SAB were measured at different separation distances under a rotational speed of 400 rpm, and the results are shown in Figure 3d. It is evident that both the short-circuit current and open-circuit voltage increase as the separation distance decreases. Specifically, the peak values of the short-circuit current and open-circuit voltage increase from 0.1 nA and 1.1 V at a 2 mm distance to 0.3 nA and 2.7 V at a 0.5 mm distance. Thus, a closer separation distance can enhance the output performance of the I-SAB. It should be noted that the separation distance should not be too small, as collisions between the electrode and triboelectric layer shorten the device’s lifespan. Considering the possible tilting of the acrylic shell under self-aligning conditions, a separation distance of 1.5 mm was determined to prevent collisions during tests within the allowable tilt range. Finally, the relationship between the output performance of the I-SAB and the number of comb–finger pairs was also analyzed and optimized. The short-circuit current and open-circuit voltage with different numbers of comb–finger pairs on the electrodes were acquired at a rotation speed of 600 rpm, and the experimental results are illustrated in Figure 3e. The results indicate that increasing the number of comb–finger pairs can result in triboelectric signals with greater signal amplitudes generated from the I-SAB. Thereby, to maximize the triboelectric signal amplitudes, we incorporated a 12-finger comb–electrode pair into the designed I-SAB.

With the optimized structural parameters, the output performance of the I-SAB under varying load resistances was also acquired under a rotational speed of 600 rpm. As shown in Figure 3f, as the load resistance increases from 100 KΩ to 9 GΩ the root-mean-square (RMS) value of the triboelectric voltage from the I-SAB increases from 0.3 V to 1.6 V, while the RMS value of the triboelectric current decreases from 0.3 nA to 0.012 A. As a result, a peak power of 0.15 nW is achieved at a load resistance of 5 MΩ.

## 3. Self-Sensing Applications

**Figure 4:**
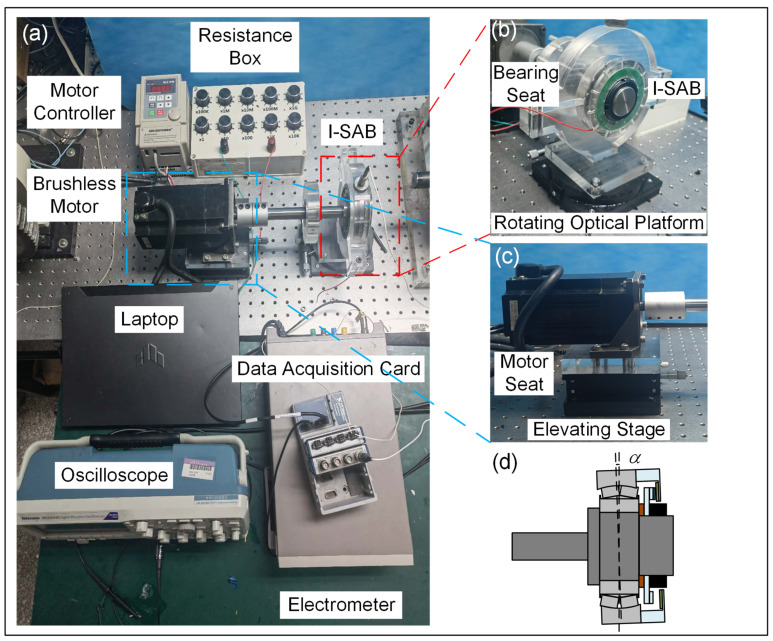
(**a**) Photograph of test bench; (**b**) structure of bearing seat; (**c**) installation of motor; (**d**) bias angle applied to I-SAB.

The newly designed I-SAB embedded with a TENG is an intelligent bearing with multiple functionalities. To illustrate its self-sensing performance, we constructed the test bench shown in Figure 4a. During the experiment, the triboelectric voltage signal was detected using a Tektronix DPO-2024B Digital Phosphor Oscilloscope (Tektronix, Portland, OR, USA), while the triboelectric current signal was measured using a Keithley 6514 System Electrometer (Tektronix, Portland, OR, USA). All data were collected with an NI Data Acquisition Card and recorded on an ASUS laptop. The inner ring of the I-SAB was fastened to a stepped shaft and connected to a brushless motor (AM-370L, Adlee Servo Motor Manufacturer, Taichung City, Taiwan), and the motor’s rotational speed was adjusted using a BL2-IPM controller.

As shown in Figure 4b, the I-SAB was mounted on a specially designed acrylic bearing seat, which was fixed to an RSP125-L rotational optical platform. The rotational optical platform can be adjusted to tilt the bearing seat and simulate the application of different bias angles to the I-SAB. Specifically, the outer ring of the I-SAB was secured to the bearing seat and could be tilted when the optical platform was adjusted, as illustrated in Figure 4d. Meanwhile, the inner ring of the I-SAB remained fixed to the rigid stepped shaft, ensuring that it was unaffected by the adjustments to the outer ring. This setup can simulate a controllable bias angle between the inner and outer ring of the I-SAB. For the adjustment of coaxiality, the motor was also fixed onto another LZ125-2-N, elevating the motor seat using an optical stage (shown as Figure 4c). This delivered one translational degree of freedom along the Z-axis. Additionally, the motor’s positioning hole diameter was 0.3 mm greater than tthe screw diameter, providing two translational degrees of freedom in the horizontal plane (X and Y) and a small rotational degree of freedom, enabling fine angular adjustments within roughly ± 0.5°. This design grants the motor three translational degrees of freedom and one rotational degree of freedom, allowing for the precise alignment of its axis with the bearing’s axis. Once aligned, the limit screws are tightened, and we use metal flat washers to minimize the load on the hole walls and to ensure the stability of the entire test bench. It is worthy of note that this configuration only ensures stability at relatively low speeds, restricting the rotational speeds in this study to under 1000 rpm.

### 3.1. Rotational Speed Sensing

**Figure 5:**
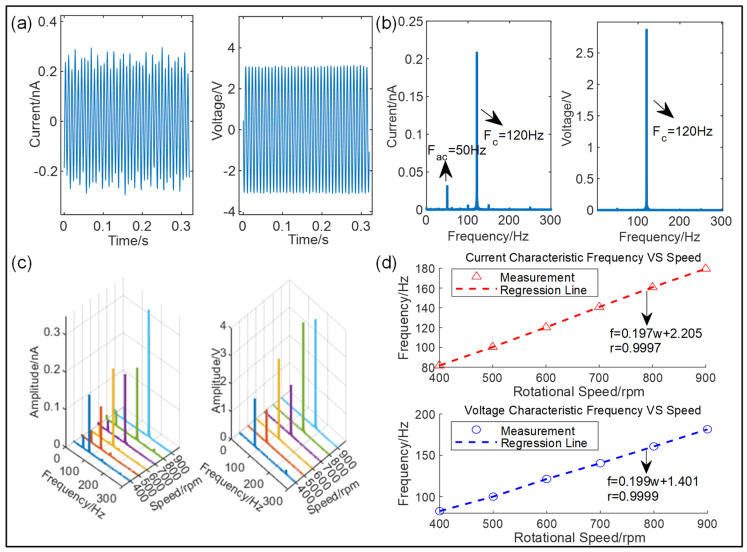
(**a**) Voltage and current signals under a rotational speed of 600 rpm; (**b**) spectra of voltage and current signals; (**c**) a waterfall plot of voltage and current signals; (**d**) a linear relationship between the prominent frequency and rotational speed.

One key functionality of the newly designed I-SAB is its self-sensing capability in terms of rotational speed. The waveforms of the open-circuit voltage and short-circuit current from the I-SAB at a rotational speed of 600 rpm are displayed in Figure 5a, and their corresponding spectra are presented in Figure 5b. The spectra show that both the triboelectric current and voltage signals have a prominent frequency of 120 Hz. Theoretically, the prominent frequency $f_{s}$ of the triboelectric signal of the TENG can be expressed using the number of comb–finger pairs *N* and the rotational frequency $f_{r}$ as follows:

(1) $$f_{s}=Nf_{r}/60$$

In our experiment, the number of comb–finger pairs *N* is 12; thus, the theoretical signal frequency corresponding to a rotational speed of 600 rpm should be 120 Hz, which is consistent with the experimental result. To further explore the relationship between the signal frequency and rotational speed, we acquired 20 s long triboelectric current and voltage signals emitted from the I-SAB at various speeds ranging from 400 rpm to 900 rpm. Furthermore, the fast Fourier transform (FFT) algorithm was applied for signal analysis to investigate the characteristic frequencies, as shown in Figure 5c. Figure 5d illustrates the relationship between the characteristic frequencies of the signals and the rotational speed. From these results, we found a strong linear relationship between the prominent signal frequency and the rotational speed. After calculation, the linear correlation coefficients for the current and voltage signals were 0.9997 and 0.9999, respectively, indicating that the prominent frequency of triboelectric signals can be an excellent indicator for the self-sensing and measurement of the rotational speed. The slopes of the linear regression lines for the current and voltage signals were calculated to be 0.197 and 0.199, respectively, which are very close to the theoretical value given by Equation (3), namely, 0.2.

### 3.2. Bias Angle Monitoring and Sensing Performance Optimization

**Figure 6:**
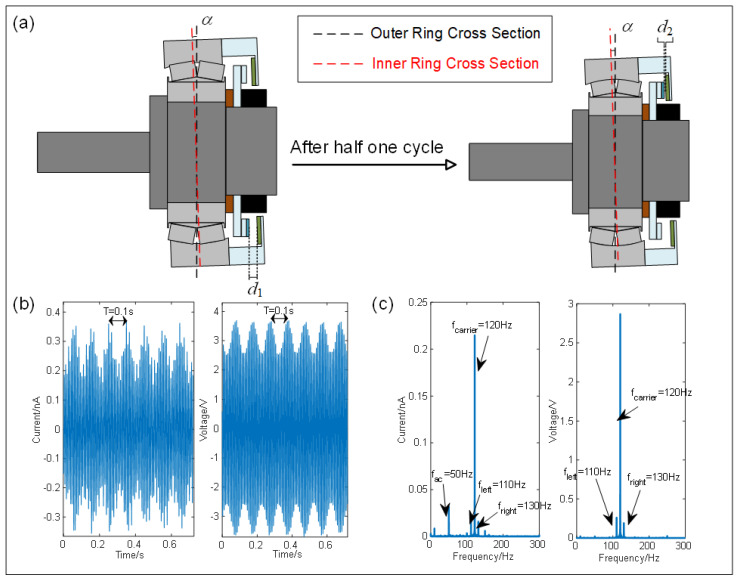
(**a**) Illustrations of the bias angle and the triboelectric–electrode distance during one half rotation; (**b**) the waveform of the triboelectric current and voltage signals of the I-SAB with a bias angle of 0.6° under a rotational speed of 600 rpm; (**c**) spectra of the triboelectric current and voltage signals.

Another key functionality of the I-SAB is its self-sensing capability to monitor the bias angle. An illustration of the bias angle and varying triboelectric–electrode distances in the operating I-SAB is depicted in Figure 6a. When a bias angle is introduced to the self-aligning bearing, the separation distance between the flannelette triboelectric layer and the electrode varies at different circumferential positions. The distance between the triboelectric layer and electrode during operation can be formulated as follows:

(2) $$d(t)=d_{0}+Rsin(\alpha )sin(\omega t+\varphi )$$

where $d_{0}$ represents the initial separation distance between the triboelectric layer and the electrode in the scenario of zero bias angle, *R* denotes the distance from the center of the triboelectric layer to the geometric center of the acrylic disk, α is the bias angle, ω is the rotational speed, and φ is the initial phase of rotation. Supposing that the self-aligning bearing (21308CA/W33 model) has a nominal maximum bias angle limitation of 1°, Equation (2) can be approximated as follows:

(3) $$d(t)\approx d_{0}+R\alpha sin(\omega t+\varphi )$$

From Equation (3), it is evident that the distance between the triboelectric layer surface and the electrode varies during rotational motions. The induced charge on the electrode decreases with an increasing triboelectric–electrode distance, leading to a reduction in the transferred charge. Conversely, the transferred charge increases as the triboelectric–electrode distance decreases. The variations in the triboelectric–electrode distance are more significant when the bias angle of the I-SAB increases. Given that the distance between the triboelectric layer and the electrode layer varies periodically at an angular frequency of ω, the output signals exhibit a sinusoidal envelope, modulated by this periodic variation. The modulation of the signals’ envelopes can be expressed via the following equations:

(4) $$Q_{i}=\int \int \varepsilon_{0}E(x,y,d(t))cos(\theta_{n})dxdy$$

(5) $$I_{sc}=\frac{Q_{i}}{∆t}=\frac{\int \int \varepsilon_{0}E(x,y,d(t))cos(\theta_{n})dxdy}{∆t}$$

(6) $$V_{oc}=\frac{Q_{i}}{∆t}Z=\frac{\int \int \varepsilon_{0}ZE(x,y,d(t))cos(\theta_{n})dxdy}{∆t}$$

where $Q_{i}$ represents the induced charge accumulated on the electrode, E(x,y,θ) is the electric field strength at a point on the electrode surface with the location of (*x*,*y*), d(t) denotes the triboelectric–electrode distance, $\varepsilon_{0}$ is the dielectric constant of the vacuum, $\theta_{n}$ is the angle between the electric field and the electrode surface, ∆t is the time duration of one rotation cycle, and Z is the internal impedance of the I-SAB. Due to the fringe effects of the electric field, the strength of the electric field on the electrode surface decreases with increasing triboelectric–electrode distance. Thus, a reduction in the triboelectric–electrode distance will increase the induced charge density. As a result, a larger bias angle will result in more significant variations in the triboelectric–electrode distance, thus leading to both an increase in voltage variation and a greater envelope amplitude on the triboelectric voltage signal. The analysis of the envelope of output triboelectric signals can provide critical insights into bias angle monitoring.

Figure 6b exhibits the waveforms of the triboelectric current and voltage signals emitted from the designed I-SAB with a bias angle of 0.6° at a speed of 600 rpm, with their corresponding spectra presented in Figure 6c. The clear periodic envelopes can be identified in both the triboelectric current and voltage signals. These envelopes have a period of approximately 0.1 s, which corresponds to one rotation period. Theoretically, the voltage signal shown in the figure can be regarded as an amplitude modulation (AM) signal, as shown in the following equations:

(7) $$V(t)=(V_{0}+Acos(2\pi f_{r}t))cos(2\pi f_{s}t)$$

(8) $$V(t)=V_{0}cos(2\pi f_{s}t)+\frac{A}{2}cos(2\pi (f_{r}-f_{s}))+\frac{A}{2}cos(2\pi (f_{r}+f_{s}))$$

where $V_{0}$ is the carrier amplitude, A denotes the envelope amplitude, $f_{r}$ represents the rotational frequency, and $f_{s}$ is the carrier frequency ($f_{r}$<<$f_{s}$). Similarly, the triboelectric current signal can also be rewritten in the same form. Working from the equivalent Equation (8), it can be stated that the three prominent frequencies in the triboelectric signals are $f_{s}$, $f_{s}-f_{r}$, and $f_{s}+f_{r}$. In the case of the I-SAB with a bias angle of 0.5° under a rotational speed of 600 rpm, the three prominent frequencies of the triboelectric signals are 110, 120, and 130 Hz, which are clearly identified in the spectra. However, there exists an apparent difference between the current and voltage envelope waveforms: only the voltage envelope is perfectly sinusoidal. The current spectra show a noticeable 50 Hz ambient interference, namely, the power frequency, which may seriously affect the monitoring of the bias angle. To ensure reliability, the triboelectric voltage signal was analyzed via the bias angle monitoring using the I-SAB.

**Figure 7:**
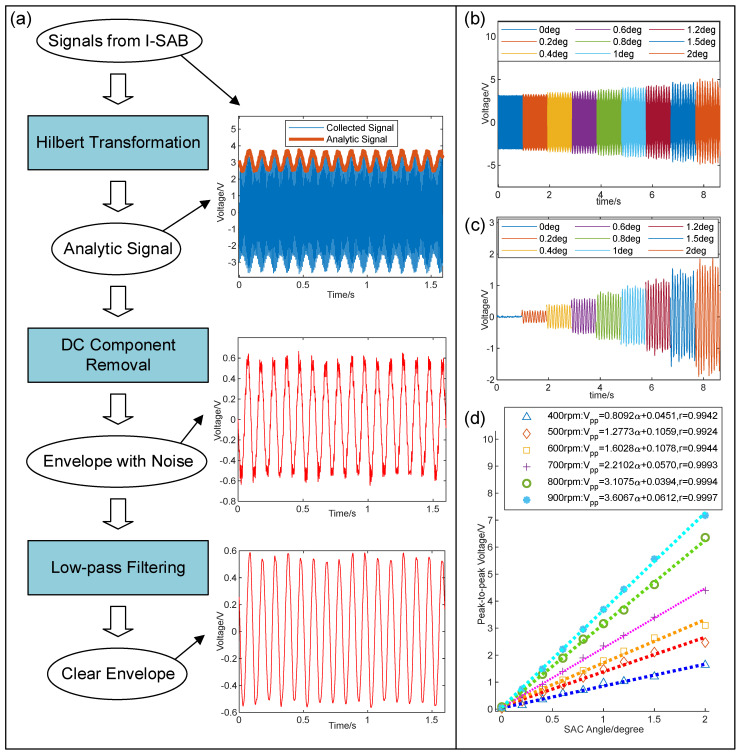
(**a**) Signal processing procedure; (**b**) voltage signals with different bias angles under speed of 600 rpm; (**c**) demodulated envelope signals with different bias angles under speed of 600 rpm; (**d**) relationship between peak-to-peak value of envelope signal and bias angle at various rotational speeds.

To analyze the envelope of the triboelectric voltage signal, the signal processing procedure was designed, as shown in Figure 7a. The Hilbert transform is a robust and computationally efficient algorithm used for analyzing the instantaneous amplitude of non-stationary signals [24], including the triboelectric voltages generated by the proposed I-SAB. In this study, the Hilbert transform was utilized to extract the envelope of the voltage signals. Firstly, the Hilbert transformation, defined in Equation (9), was applied to the voltage signal V(t), which yields the imaginary part $\hat{V(t)}$ of the analytic signal:

(9) $$\hat{V(t)}=H(V(t))=V(t)\;*\;\frac{1}{\pi t}$$

The analytical signal $\tilde{V(t)}$ of the voltage signal V(t) is defined as follows:

(10) $$\tilde{V(t)}=V(t)+j\hat{V(t)}\;$$

Since $f_{s}=12f_{r}$ in Equation (7), which ensures no overlap between the spectrum of the carrier and the envelope, the Hilbert transformation of the triboelectric voltage signal can be written in the following form according to the Bedrosian Theorem [25]:

(11) $$\hat{V(t)}=H((V_{0}+Acos(2\pi f_{r}t))cos(2\pi f_{s}t))=(V_{0}+Acos(2\pi f_{r}t))H(cos(2\pi f_{s}t))$$

By substituting Equations (7) and (11) into Equation (10), the following equation can be yielded:

(12) $$\tilde{V(t)}=(V_{0}+Acos(2\pi f_{r}t))(cos(2\pi f_{s}t)+jsin(2\pi f_{s}t))\;$$

Furthermore, the envelope amplitude of the analytic signal can be written as follows:

(13) $$\left|\tilde{V(t)}|=V_{0}+Acos(2\pi f_{r}t\right)$$

According to Equation (13), the magnitude of the analytic signal can directly represent the envelope amplitude when there is a DC component that can be easily removed by subtracting the mean value.

As illustrated in Figure 7a, the presence of high-frequency noise can still be observed in the envelope signal with the removal of the DC component. Therefore, the low-pass filtering operation was further applied to mitigate the noise interference. Eventually, the extracted envelope signal exhibited a perfect sinusoidal form, as illustrated in Figure 7a. To further explore the influence of the bias angle on the envelope signal, we acquired triboelectric voltage signals with various bias angles. The resulting original waveforms are shown in Figure 7b, with the corresponding demodulated envelope signals shown in Figure 7c. It is evident that the envelope amplitude increases with the increasing bias angle, showing that the AM voltage signal from the I-SAB can serve as an excellent indicator of the self-sensing of the I-SAB’s bias angle. Figure 7d illustrates the relationship between the peak-to-peak value of the envelope voltage signal and the bias angle at various rotational speeds. This was determined using the linear regression method. The results in Figure 7d show that the sensitivity of the peak-to-peak voltage to the bias angle increases with the rotational speed, rising from 0.8092 V/deg at 400 rpm to 3.6067 V/deg at 900 rpm. In addition, the results also reveal a strong linear correlation between the peak-to-peak value of the envelope voltage and bias angle, with the Pearson correlation coefficients all exceeding 0.99. Eventually, to determine the relationship between the line slope K and the rotational speed ω, the linear regression method was applied. The formulation obtained is as follows:

(14) K=0.0057ω−1.6279

The Pearson correlation coefficient of the linear regression in Equation (14) is 0.9894, which indicates the existence of a strong linear relationship between the sensing sensitivity and rotational speed. According to Equation (14), we can rewrite the relationship between the bias angle and peak-to-peak value V_pp_ of the voltage envelope as follows:

(15) $$\hat{\alpha }=\frac{V_{pp}}{\left(0.0057\omega -1.6279\right)}$$

where $\hat{\alpha }$ denotes the estimated bias angle. To demonstrate the accuracy of the bias angle monitoring, we calculated the deviation value and deviation rate under different bias angles, given by Equation (15), using the following formulae:

(16) $$Deviation=|\hat{\alpha }-\alpha |$$

(17) $$Deviation\;Rate=\frac{Deviation}{\alpha }\times 100\%$$

**Figure 8:**
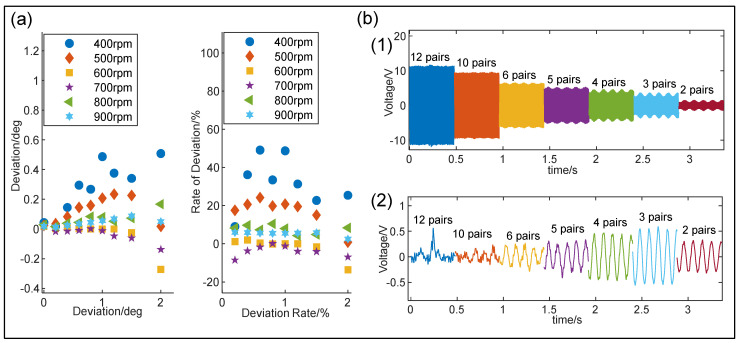
(**a**) Deviation and deviation rate given by the linear regression algorithm; (**b**) voltage (1) and envelope signals (2) given by the I-SABs containing different numbers of flannelette triboelectric layers.

The calculated results are shown in Figure 8a, indicating that the deviation and deviation rates of bias angle sensing are the most pronounced at the rotational speed of 400 rpm; meanwhile, the deviation rate reaches its highest value of approximately 50% at bias angles of 0.6° and 1°. However, as the rotational speed increases, both the deviation and deviation rate decrease significantly. When the rotational speed exceeds 600 rpm, the deviation rate can be maintained within the range of ±10%.

To maximize the voltage signal amplitude and improve the capability of bias angle monitoring, the number of dielectric patches was further optimized. Specifically, different numbers of flannelette triboelectric layers were adhered to the acrylic disk and voltage signals were acquired from the designed I-SAB with a bias angle of 0.6° at a rotational speed of 600 rpm. As shown in Figure 8b, the disk with 12 dielectric patches produced a maximum voltage peak-to-peak value of 22 V; however, no recognizable envelope exists under this condition. With a decreasing number of dielectric patches, the envelope amplitude increases and reaches a maximum peak-to-peak value of 1.07 V with three dielectric patches, although the voltage signal amplitude decreases continuously. The main reason for this is that reducing the number of dielectric patches decreases the total charge induced on the electrode, but it increases the variations in the induced charges during each rotation cycle, leading to an increase in the envelope amplitude. Ultimately, to yield the maximum envelope amplitude and a superior capability in terms of bias angle monitoring, three pairs of flannelette triboelectric layers were adhered to the acrylic disk.

## 4. Fault Diagnosis Application

Due to the harsh working conditions, the key components of self-aligning bearings are prone to faults after prolonged operation; therefore, it is necessary to monitor and diagnose the condition of the bearings. To address this issue, we explored the usage of triboelectric signals emitted from the newly designed I-SAB for self-powered fault diagnosis. In detail, the output triboelectric signals emitted from the I-SAB were collected under various conditions including ones with no fault, an inner ring fault, an outer ring fault, a roller fault, and a rotor imbalance fault. All datasets were acquired across six different rotational speeds. Moreover, we developed an end-to-end deep learning model, namely, the Multi-Scale Discrimination Network (MSDN), based on the ResNet18 architecture, to implement the automatic fault diagnosis application. The developed MSDN is a promising method used to extract the hidden fault features embedded in the triboelectric signals emitted from our designed I-SAB.

**Figure 9:**
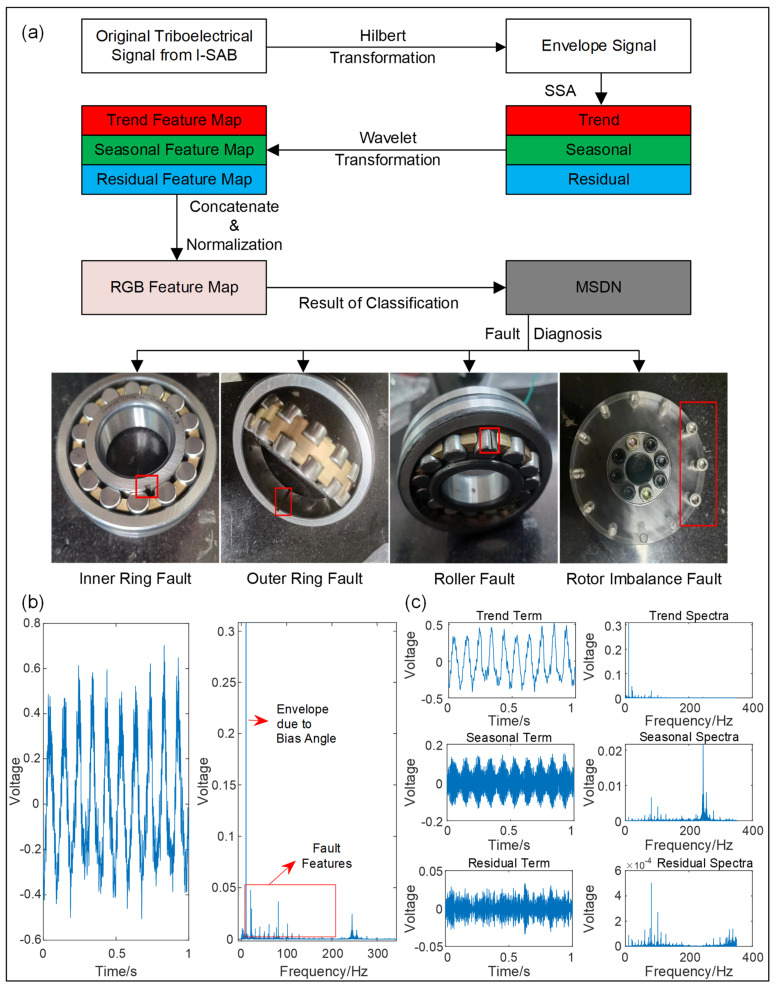
(**a**) The data processing flowchart; (**b**) the waveform and spectrum of the voltage envelope signal under the health condition of an inner ring fault and a rotational speed of 600 rpm; (**c**) waveforms and spectra for the trend term, seasonal term, and residual term given by the singular spectrum analysis.

A data processing flowchart showing the self-powered fault diagnosis application is presented in Figure 9a. Since bearing fault features are modulated onto a carrier signal through amplitude modulation [23], the envelope signals are first obtained through the Hilbert transform for feature enhancement. Taking the triboelectric voltage signals under the condition of an inner ring fault at a rotational speed of 600 rpm as an example, the waveforms of the envelope signal and the corresponding spectrum are displayed in Figure 9b. The envelope spectrum in Figure 9b indicates that the frequency components related to fault features are overshadowed by the sinusoidal component produced by the bias angle. To extract and enhance the weak fault features related to different health conditions, singular spectrum analysis (SSA) [23] was further applied to the envelope signal. This SSA technique decomposes the envelope signal into three different scales—the trend term, the seasonal term, and the residual term—thus enabling feature extraction in these three scales. As for the implementation details of SSA, firstly, the envelope signal *x* with a length of *N* is embedded into a trajectory matrix using slide-window sampling with the window length *L*. This is performed as follows:

(18) $$X=\left[\begin{array}{ccc} \begin{array}{c} x\;\left[0\right]\\ \vdots \\ x[L-1] \end{array} & \begin{array}{c} x\;\left[1\right]\\ \vdots \\ x[L] \end{array} & \begin{array}{cc} \cdots & \begin{array}{c} x[N-L]\\ \vdots \\ x[N-1] \end{array} \end{array} \end{array}\right]$$

Secondly, by applying the singular value decomposition (SVD) to the trajectory matrix X, we obtain the following:

(19) $$X=U\Sigma V^{T}=\;\left[{\overset{⃑}{u}}_{0},{\overset{⃑}{u}}_{1,}...,{\overset{⃑}{u}}_{i},...,{\overset{⃑}{u}}_{L-1}\right]\left[\begin{array}{cccc} \sqrt{\lambda_{0}} & 0 & 0 & \begin{array}{cc} 0 & \begin{array}{cc} \cdots & 0 \end{array} \end{array}\\ 0 & \ddots & 0 & \begin{array}{cc} 0 & \begin{array}{cc} \cdots & 0 \end{array} \end{array}\\ 0 & 0 & \sqrt{\lambda_{L-1}} & \begin{array}{cc} 0 & \begin{array}{cc} \cdots & 0 \end{array} \end{array} \end{array}\right]{\left[\begin{array}{c} {\overset{⃑}{v}}_{0}\\ \vdots \\ {\overset{⃑}{v}}_{N-L} \end{array}\right]}_{\left(N-L+1)\times (N-L+1\right)}$$

where ${\overset{⃑}{u}}_{i}\in R^{L\times 1}$ is the $i^{th}$ column vector in orthogonal matrix U, $\sqrt{\lambda_{j}}\in R$ is the $j^{th}$ eigenvalue, and ${\overset{⃑}{v}}_{k}\in R^{1\times (N-L+1)}$ is the $k^{th}$ row vector in the orthogonal matrix $V^{T}$. The $i^{th}$ component of the trajectory matrix can also be represented as follows:

(20) $$X^{i}=\sqrt{\lambda_{i}}{\overset{⃑}{u}}_{i}{\overset{⃑}{v}}_{i},\;i\in [0,L-1]$$

Thirdly, the $i^{th}$ signal component, ${\hat{x}}^{i}$, is recovered from the corresponding component matrix by calculating the average of the reverse diagonal elements:

(21) $${\hat{x}}^{i}[d]=\frac{1}{n_{d}}\sum_{m+n=d}X^{i}[m,n]$$

where *n_d_* denotes the element number in the *d* ^th^ reverse diagonal. In our study, *N* = 125,000 and *L* = 10. To reconstruct the signal components in the three different scales, we categorized the components into three groups based on their eigenvalue magnitude and then superposed the discrete sequences within each group. The reconstructed sequences in three different scales and their spectrum are displayed in Figure 9c.

As deep learning models require a large sample size, data augmentation was conducted using sliding window sampling; this enables deep learning models to learn local invariant features and enhances the model’s generalization capability. The sample number obtained from each component of the fault signal, S, can be calculated as follows:

(22) $$S=\frac{N-M}{Slide}+1$$

where *N* is the length of the signal components, Slide refers to the overlap length, and *M* is the window length. The wavelet transformation effectively captures local features such as transients, discontinuities, and singularities [26]. In this study, the Morlet wavelet was selected as the mother wavelet for the wavelet transformation, as formulated below:

(23) $$\psi (t)=e^{j\omega_{0}t}e^{-\frac{t^{2}}{2}},t\epsilon [-4,4]$$

where $\omega_{0}$ is the center frequency of the mother wavelet. The Morlet wavelet can be viewed as a complex sinusoidal wave modulated by a Gaussian envelope. This unique structure enables the Morlet wavelet to exhibit high sensitivity to time-variant features. Consequently, the continuous wavelet transformation based on the Morlet wavelet was further applied to the signal components to obtain component spectrograms at three different scales. The component spectrograms in the three scales were then concatenated into three-channel feature maps that were subsequently stored as RGB jpeg images. It is worthy of note that channel normalization was also applied to each feature map before saving. These RGB images were then fed into the deep learning models for end-to-end fault diagnosis.

**Figure 10:**
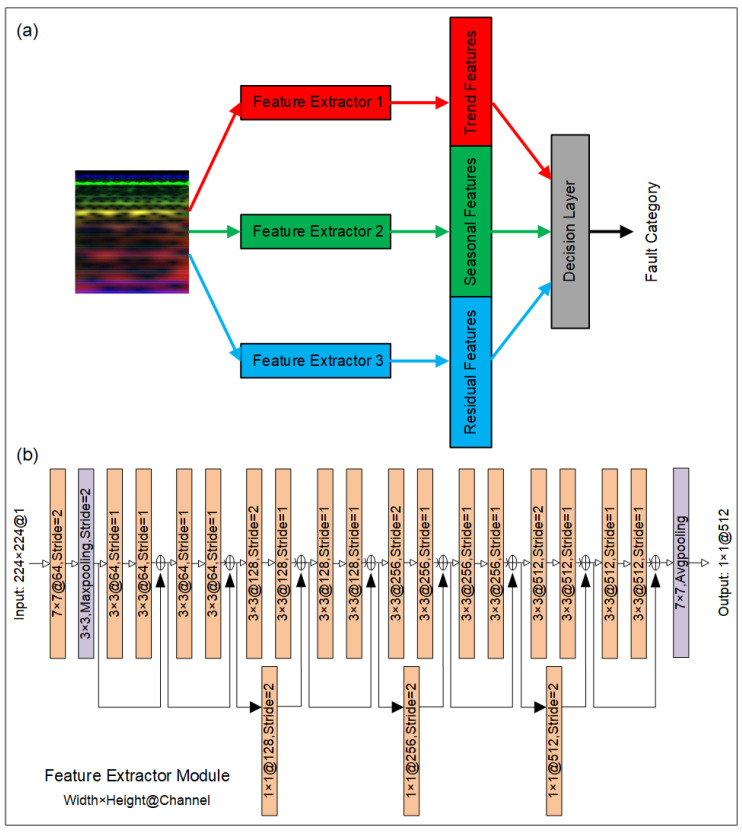
(**a**) Structure of MSDN; (**b**) architecture of feature extractor module based on ResNet18.

To address the task of end-to-end fault diagnosis with weak fault features in the RGB images of triboelectric signals, we developed a novel deep learning model, the MSDN. The structure of the MSDN is shown in Figure 10a. The developed MSDN model consists of three feature extractor modules and a decision layer, aiming to extract weak fault features from the perspective of time–frequency spectrograms and achieve fault diagnosis with high diagnosis accuracy. To accurately extract fault features at different scales, ResNet18 [27] was selected as the baseline of the feature extractor modules in our developed MSDN model. The architecture of the ResNet18-based feature extractor module is shown in Figure 10b. It is worthy of note that the features from different channels in traditional convolution layers are summed up after the convolution operations; hence, the weak features would be overshadowed by the stronger ones. As a result of the premature feature mixing, some important features disappear due to this inter-channel superposition. To mitigate this, we introduced three independent feature extractor modules to process the three-channel spectrogram separately at each scale. This strategy ensures that each feature extractor can specially extract features for one single feature map. The features from one scale do not sum up with those from other scales until reaching the decision layer, where the final feature fusion based on the fully connected layer occurs. This delayed fusion helps the model to extract weak fault features from each channel in turn [28]. The fused features are finally reduced to a 5D vector, which is then input into a SoftMax classifier for fault classification.

In our fault diagnosis experiments, the triboelectric signals emitted from the I-SAB were sampled in each condition for 40 s, and the total length of these signals was 125,000. The sliding window length *M* was 3125, and the Slide was 625. Both of these values were further applied to segment the components into smaller pieces. According to Equation (22), the number of samples obtained from one scale under one working condition was 196. Since the complete dataset consists of samples from six different rotational speeds (400 rpm, 500 rpm, 600 rpm, 700 rpm, 800 rpm, and 900 rpm), each with five different working conditions, the complete dataset consisted of 5880 samples. In our experiment, 60% of the samples from each working condition were used in the training set, 20% were used in the validation set, and the remaining 20% were used in the testing set. To avoid the overfitting issue, the learning rate was set to 10^−5^, and the stochastic gradient descent optimizer was used for model training. The loss function was cross-entropy, and deep learning models were built using Pytorch 1.12.0 on a Python 3.9 interpreter.

**Figure 11:**
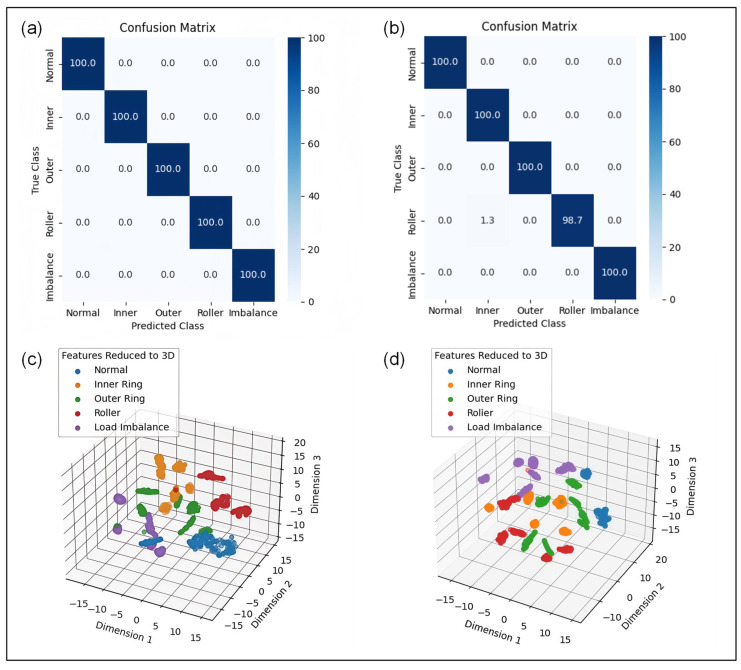
Fault diagnostic results of the proposed MSDN model. (**a**) Optimal confusion matrix using triboelectric voltage signals; (**b**) optimal confusion matrix using triboelectric current signals; (**c**) t-SNE feature visualization using triboelectric voltage signals; (**d**) t-SNE feature visualization using triboelectric current signals.

The fault diagnostic results of the proposed MSDN model built using triboelectric voltage and current signals emitted from the newly designed I-SAB are illustrated in Figure 11. The confusion matrix in Figure 11a demonstrates that the proposed MSDN model built using triboelectric voltage signals can exhibit superior diagnostic accuracy with a maximum level of 100%. To evaluate the robustness of the proposed model, five independent repeated experiments were carried out, resulting in a high average accuracy of 99.8% with a standard deviation of 0.23%. By contrast, the proposed MSDN model designed using the triboelectric current signals emitted from the I-SAB also yielded an optimal diagnostic accuracy of 99.7%, as shown in Figure 11b. The average accuracy derived from the triboelectric current signals emitted from the I-SAB reached 99.1% with a standard deviation of 0.71%. Additionally, the t-distributed stochastic neighbor embedding (t-SNE) algorithm was further applied to visualize the features of the final layer in the proposed MSDN model. The t-SNE feature visualization results shown in Figure 11c,d indicate that the output features of the MSDN using the triboelectric voltage and current signals emitted from the I-SAB can be perfectly separated, thus further validating the robustness of the proposed MSDN model.

**Figure 12:**
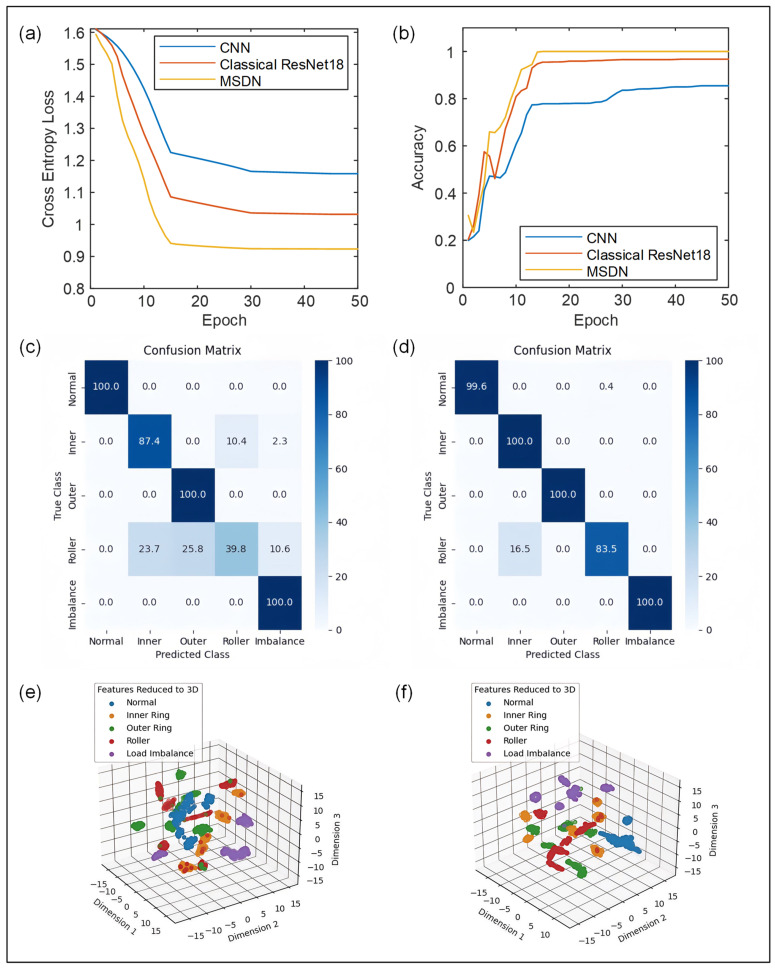
(**a**) The variation in training losses seen for three different methods. (**b**) The variation in training accuracies seen for three different methods. (**c**) The confusion matrix for CNN using triboelectric voltage signals, where accuracy = 85.4%. (**d**) The confusion matrix for ResNet18 using triboelectric voltage signals, where accuracy = 96.6%. (**e**) T-SNE feature visualization for the output features of CNN. (**f**) T-SNE feature visualization for output features of ResNet18.

In order to compare the newly proposed MSDN with classical deep learning models, the diagnostic performances of the CNN and ResNet18 using triboelectric voltage signals, including the training loss, confusion matrix, and feature visualization results, were further comprehensively analyzed. The training loss and accuracy during the training stage for the three different methods are displayed in Figure 12a and Figure 12b, respectively. It can be observed that, after 15 training epochs, the best accuracy of the MSDN was maintained at 100%. To verify the possibility of overfitting, the proposed MSDN model was further examined on the validation set and the optimal diagnostic accuracy also reached 100%. These results confirm that the proposed MSDN model has a higher convergence speed and superior diagnostic accuracy compared to classical CNN and ResNet18 models, whose average diagnostic accuracies are 85.4% and 96.6%, respectively. Furthermore, the confusion matrices for CNN and ResNet18 are shown in Figure 12c,d, which indicate that neither CNN nor ResNet18 could perfectly distinguish between inner ring faults and roller faults. In addition, the t-SNE feature visualization results for the CNN and ResNet18 in Figure 12e,f show that the output features are overlapped, illustrating their weakness in terms of inter-channel feature extraction. By contrast, the MSDN has the least feature overlaps, indicating that it has excellent potential in feature separation. These comparison results solidly confirmed that the proposed MSDN model, which uses the triboelectric signals emitted from the I-SAB, exhibits superior convergence speed, diagnostic accuracy, and feature separation capability for self-powered fault diagnosis. Moreover, with the aid of the proposed MSDN model, triboelectric signals emitted from the I-SAB can be used as accurate and reliable indicators in self-powered fault diagnosis.

## 5. Conclusions

In this study, a novel self-powered, intelligent, and self-aligning bearing (I-SAB) embedded with a triboelectric nanogenerator (TENG) was designed and implemented. This innovative smart bearing demonstrated extremely high accuracy in rotational speed sensing. Most notably, the newly designed I-SAB performed the first ever example of self-powered bias angle monitoring in a rotating machinery system. Benefiting from its unique design, the triboelectric voltage signal from the I-SAB exhibits an amplitude-modulated envelope, which occurs when a bias angle is present in the designed SAB device. A strong linear relationship was observed between the envelope amplitude of the triboelectric voltage signals and the bias angle. Moreover, the sensitivity of the bias angle sensing exhibited a linear correlation with the rotational speed. These two strong linearities enabled the proposed I-SAB to achieve self-powered and reliable bias angle sensing. To facilitate fault diagnosis using the I-SAB, a new neural network framework, namely, the Multi-Scale Discrimination Network (MSDN), was developed for weak feature extraction and self-powered fault diagnosis. The proposed MSDN trained using the triboelectric voltage and current signals yielded excellent average diagnosis accuracies of 99.8% and 99.1%, respectively, surpassing traditional deep learning models, including CNN and ResNet18. In summary, the newly designed I-SAB has shown considerable potential for self-powered rotational speed sensing, reliable bias angle monitoring, and accurate fault diagnosis. Furthermore, this work pioneers self-powered bias angle monitoring and fault diagnosis in rotating machinery systems supported by self-aligning bearings.

However, due to equipment limitations, the performance of the I-SAB under variable temperatures and high-speed conditions and during long-term operation was not investigated in this study. In the future, we plan to examine the performance of the I-SAB under extreme conditions and further optimize its design to ensure adaptability to harsh environments and to fault diagnosis applications under varying speed conditions.

## Data Availability

Data will be provided upon request.
